# Plastic bronchitis linked to human bocavirus 1 identified through high-throughput next-generation sequencing: A case report

**DOI:** 10.1097/MD.0000000000039361

**Published:** 2024-09-06

**Authors:** Xiumin Zhang, Jing Zhao

**Affiliations:** a Department of Pediatric, Liaocheng People’s Hospital, Liaocheng, China.

**Keywords:** bronchoscopy, human bocavirus 1, plastic bronchitis

## Abstract

**Background:**

Plastic bronchitis (PB) is an uncommon and severe acute respiratory ailment characterized by the formation of casts in the trachea or bronchial tree. Some instances have been linked to human bocavirus (HBoV) infections.

**Case presentation:**

In this report, we present a case of PB secondary to HBoV1 infection in a previously healthy pediatric patient. A 17-month-old male was admitted due to respiratory distress following 2 days of cough and fever. A preadmission chest X-ray revealed atelectasis of the left lung. Emergency electronic bronchoscopy and foreign body forceps were employed to remove casts, leading to improved breathing. High-throughput next-generation sequencing detected only HBoV1. A subsequent electronic bronchoscopy 2 days later showed no casts.

**Conclusions:**

PB associated with HBoV1 infection should be considered in children experiencing acute respiratory distress, and a second bronchoscopy intervention may not be necessary in cases related to HBoV1.

## 1. Introduction

Plastic bronchitis (PB) presents a serious respiratory challenge due to airway obstruction from the formation of mucus plugs in the bronchi. Some reported cases indicate a connection between human bocavirus (HBoV1) and PB. HBoV1 is a small, icosahedral, linear, non-enveloped, single-stranded DNA virus ranging in size from 18 to 26 nm.^[1]^ It is primarily found in respiratory tract samples and can cause mild to severe upper and lower respiratory tract infections, including life-threatening respiratory illness in children.^[2]^ The pathogenesis of HBoV1 is not well-understood, and there is currently no clinically approved specific treatment for HBoV1 infection. This report details the clinical features, diagnosis, and therapy for a rare case where a previously healthy boy developed PB after HBoV1 infection.

## 2. Case report

**Figure 1.**
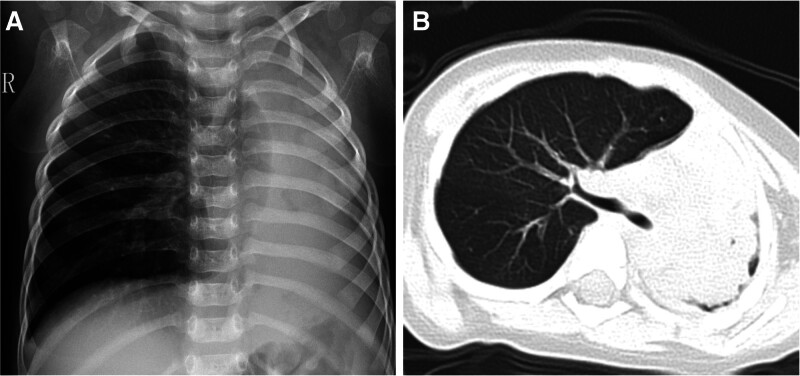
(A) Chest radiograph depicting atelectasis of the left lung; (B) chest computed tomography revealing a high-density image of the left main bronchi and atelectasis of the left lung.

A 17-month-old male, the son of a colleague, presented with respiratory distress after experiencing 2 days of cough and fever. Two weeks prior to admission, the patient had been diagnosed with an upper respiratory tract infection, exhibiting symptoms of fever, cough, and wheezing. There was no history of asthma or cardiac anomalies. Upon arrival at our department, the patient displayed tachypnea, hypoxia, a heart rate of 170 beats/min, a respiratory rate of 50 breaths/min, and a SpO2 of 86%. Examination revealed chest-wall retraction and reduced breath sounds over the left hemithorax. Chest X-ray indicated atelectasis of the left lung, while computed tomography revealed a high-density image of the left main bronchi and atelectasis of the left lung (Fig. 1). Non-invasive mechanical ventilation was promptly initiated.

**Figure 2.**
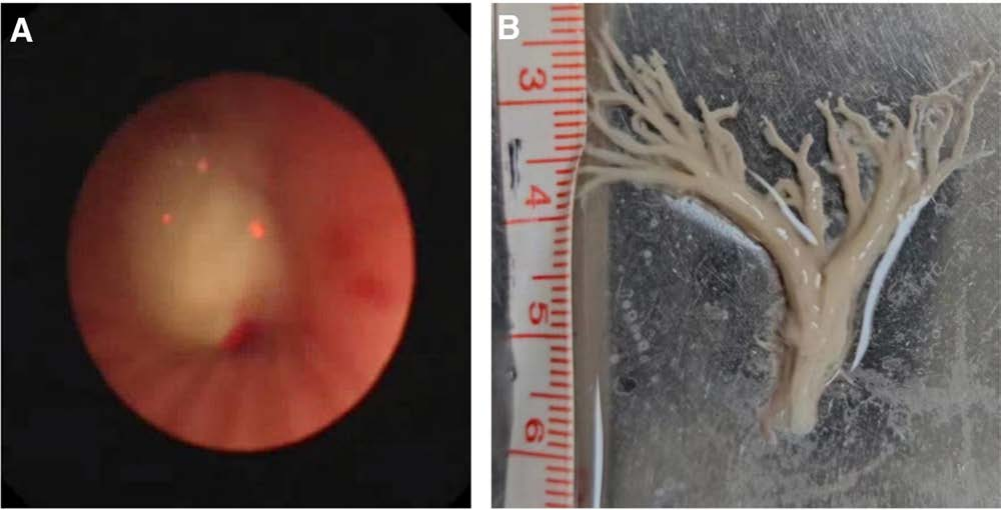
(A) Electronic bronchoscopy displaying a complete airway obstruction in the left main bronchus; (B) bronchial casts extracted from the left main stem bronchus.

**Figure 3.**
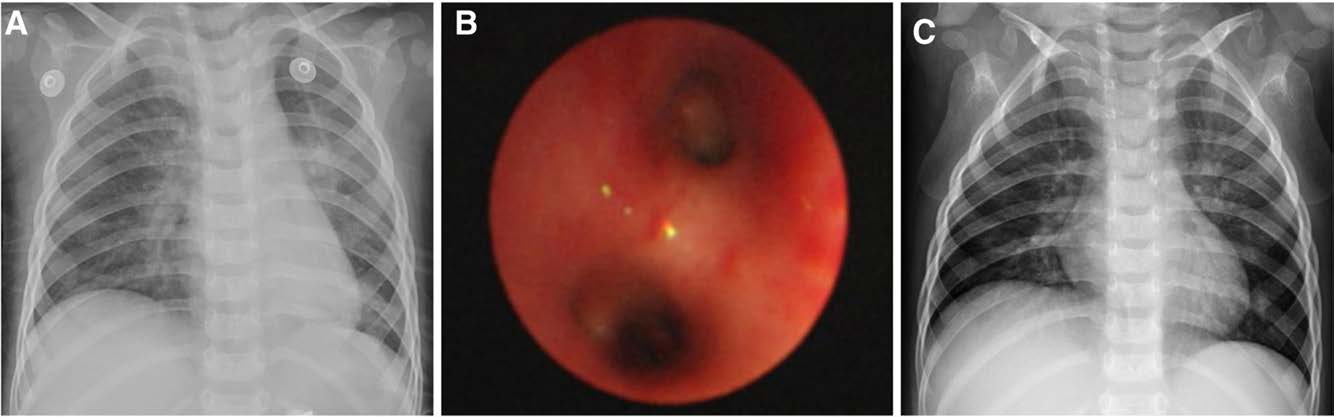
(A) Subsequent electronic bronchoscopy revealing no casts in the bronchus; (B) chest radiograph post-bronchoscopy indicating a significant improvement in atelectasis in the left lung; (C) chest radiograph displaying normal results 2 weeks after discharge.

**Figure 4.**
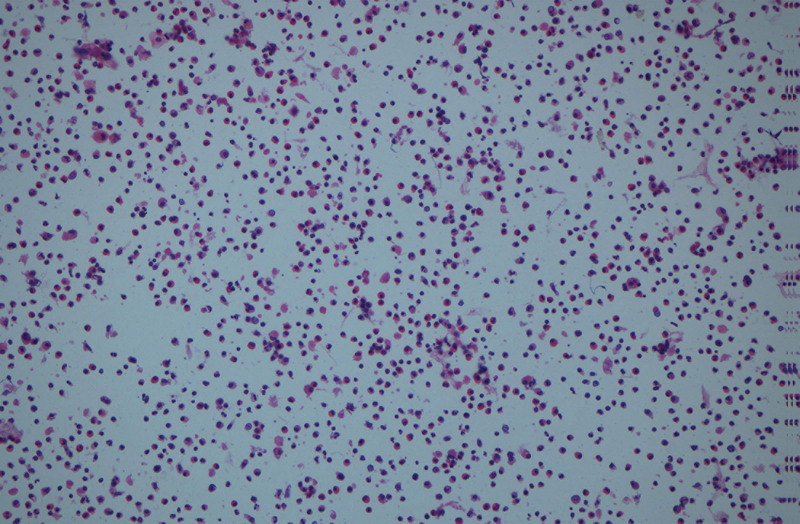
Histopathology of the casts characterized by fibrin and a substantial presence of eosinophilic granulocytes, as well as numerous neutrophils and monocytes. Moreover, there was no observation of Charcot–Leyden crystals (CLC).

Emergency electronic bronchoscopy was performed following a 4-hour period of fasting, leading to the removal of a sizable bronchial cast using electronic bronchoscopy and foreign body forceps (Fig. 2). The patient’s atelectasis significantly improved post-endoscopy (Fig. 3). High-throughput next-generation sequencing (HT-NGS) analysis of bronchial alveolar lavage fluid identified the presence of HBoV1 exclusively, with reads count of 976,068 and a concentration of 10^7^ copies/mL. Histopathological examination of the casts revealed fibrin, numerous eosinophilic granulocytes, as well as neutrophils and monocytes (Fig. 4). Blood immunoglobulin E levels were within the normal range at 87.39 IU/mL, and the eosinophil count in the patient’s blood was normal at 0.31 × 10^9^/L. Treatment included intravenous administration of 1 g/kg human gamma-globulin, methylprednisolone at 4 mg/kg/d for 4 days, and azithromycin at 10 mg/kg/d.

The child’s illness developed over a 2-day period, resulting in the formation of plastic bronchial casts. Two days later, a bronchoscopy was conducted to evaluate if there has been a recurrence of cast formation in the airways. A second electronic bronchoscopy revealed no casts (Fig. 2). The patient was discharged after 6 days, and a follow-up chest X-ray 2 weeks post-discharge displayed normal results. Six months later, we followed up with the child and found them to be in good health, with no coughing, wheezing, or breathing difficulties.

## 3. Discussion

PB is a severe respiratory disorder characterized by the presence of casts in the trachea or bronchial tree. These casts are categorized into 2 types: Type I, primarily composed of fibrin with a dense eosinophilic inflammatory infiltrate, often seen in patients with asthma, allergic diseases, and cystic fibrosis; and Type II, mainly consisting of mucin with minimal or no cellular infiltrate, occurring exclusively in children with congenital cyanotic heart disease.^[3,4]^ The diagnosis of PB relies on clinical presentation, including the expectoration of bronchial casts, along with bronchoscopic and imaging findings from computed tomography or magnetic resonance lymphangiogram images. The mechanisms underlying PB remain unclear, but genetic predisposition, increased pulmonary venous pressure, low cardiac output, mucus hyper-secretion, and inflammation leading to heightened alveolar capillary permeability are considered potential contributors.^[5]^ Various infectious agents, including HBoV1, influenza virus, adenovirus, SARS-CoV-2 virus, *Mycoplasma pneumoniae*, and opportunistic fungi, have been implicated in PB.^[4]^

HBoV1 is a member of the Parvoviridae family and was first identified in 2005. It is predominantly associated with respiratory tract infections in children. HBoV1 has been implicated in various respiratory illnesses, including bronchiolitis, pneumonia, and acute wheezing. Studies have shown a high prevalence of HBoV1 in nasopharyngeal swabs of children with respiratory symptoms, highlighting its significance as a respiratory pathogen.^[6,7]^ Recent studies have expanded our understanding of HBoV1’s pathogenesis. It has been observed that HBoV1 can cause persistent infections, and the virus can be detected in respiratory samples long after the acute phase of illness. This persistence may contribute to prolonged respiratory symptoms and complications in some patients. Moreover, HBoV1 is often found in co-infections with other respiratory viruses, which can complicate clinical presentations and outcomes.^[8]^ The link between HBoV1 and PB is not well-documented in the literature, making this case report particularly noteworthy. There have been isolated reports of PB in the context of viral infections, but comprehensive studies specifically linking HBoV1 to PB are limited. The identification of HBoV1 in this case highlights the potential role of this virus in the pathogenesis of PB, suggesting that viral infections may trigger or exacerbate the formation of bronchial casts.^[8]^

In this report, we describe a case of PB attributed to HBoV1 infection in a previously healthy child. HBoV1 was identified through HT-NGS in bronchial alveolar lavage fluid. High-throughput next-generation sequencing revolutionizes genomic research by allowing rapid and comprehensive analysis of DNA and RNA. It operates through massively parallel sequencing, where millions of fragments are sequenced simultaneously. Key principles include library preparation, sequencing-by-synthesis, and data analysis. In clinical diagnostics, HT-NGS enables precise identification of genetic mutations, aiding in personalized medicine. It is pivotal in detecting rare genetic disorders, cancers, and infectious diseases. By providing detailed genetic information, HT-NGS improves diagnostic accuracy, guides targeted therapies, and enhances our understanding of complex diseases, making it a cornerstone of modern clinical genomics. The bronchial casts, located on the left side, exhibited histopathological features consistent with Type I, comprising fibrin, numerous eosinophilic granulocytes, as well as neutrophils and monocytes. Notably, all reported cases of PB caused by HBoV have shown similar histopathological characteristics, including eosinophil-rich casts and, in 1 case, bipyramidal Charcot–Leyden crystals.^[9–12]^

Charcot–Leyden crystals are indicative of eosinophilic inflammation.^[13]^ HBoV1 infection may induce acute eosinophilic inflammation, leading to increased lymphatic permeability and triggering eosinophilic extracellular trap cell death. This process results in the formation of eosinophil extracellular traps and Charcot–Leyden crystals, contributing to the thickness and viscosity of eosinophilic material and, ultimately, bronchial cast formation.^[14]^ The patient exhibited normal levels of total immunoglobulin E, suggesting an absence of hyper-sensitivity. Interestingly, the left-sided location of the casts in our case and the previously reported cases may be associated with the drainage pattern of the thoracic duct, which typically drains into the posterior aspect of the left internal jugular and subclavian vein confluence.^[15]^ Additionally, anatomical differences, such as the longer and narrower left mainstem bronchus, could contribute to this observed asymmetry.

The therapeutic approach for HBoV1-induced PB lacks standardized guidance and relies on case reports. The primary management involves the extraction of large casts through bronchoscopy, with cryoextraction being a potentially more effective and less time-consuming option. Additional tools and interventions, including forceps, rigid bronchoscopy, inhaled and systemic corticosteroids, macrolide antibiotics, N-acetylcysteine, ambroxol, hypertonic saline, bronchodilators, sirolimus, and lymphatic ductal interventions, may be employed to facilitate bronchial cast disruption and evacuation.^[4,5,16]^ Of note, sirolimus and lymphatic interventions are relevant to Type II PB. Our case involved noninvasive mechanical ventilation for respiratory support due to respiratory distress, electronic bronchoscopy, and the use of azithromycin for its anti-inflammatory properties. Intravenous human gamma-globulin and methylprednisolone were administered post-electronic bronchoscopy. A second electronic bronchoscopy, consistent with findings by Huang et al,^[17]^ showed no mucus plugs, suggesting that a second bronchoscopy intervention may not be necessary in the HBoV1-related PB. Long-term follow-up is crucial to assess the possibility of recurrence.

In conclusion, consideration of PB is essential in children presenting with fever, coughing, wheezing, asymmetry of auscultatory changes over the lung fields, rapid progression of respiratory distress, and hypoxemia. Early recognition and prompt initiation of appropriate therapy may improve prognosis. This case report highlights the importance of considering HBoV1 in cases of acute respiratory distress in children and demonstrates the potential of HT-NGS in diagnosing such conditions. Informed consent was obtained from the patient’s parents for the publication of this report.

## Author contributions

**Conceptualization:** Xiumin Zhang.

**Data curation:** Xiumin Zhang, Jing Zhao.

**Formal analysis:** Xiumin Zhang.

**Funding acquisition:** Xiumin Zhang.

**Investigation:** Jing Zhao.

**Methodology:** Jing Zhao.

**Project administration:** Xiumin Zhang.

**Resources:** Jing Zhao.

**Software:** Xiumin Zhang.

**Supervision:** Xiumin Zhang, Jing Zhao.

**Validation:** Xiumin Zhang, Jing Zhao.

**Visualization:** Jing Zhao.

**Writing – original draft:** Xiumin Zhang, Jing Zhao.

**Writing – review & editing:** Xiumin Zhang, Jing Zhao.
